# Editorial: Improving understanding and treatment of peripheral positional vertigo (PPV)

**DOI:** 10.3389/fneur.2025.1773353

**Published:** 2026-01-28

**Authors:** Anita Bhandari, Francisco Carlos Zuma E Maia, Norberto Martinez, Michael Strupp

**Affiliations:** 1 NeuroEquilibrium Diagnostic Systems Pvt Ltd., Jaipur, India; 2 Department of Otorhinolaryngology, Mahatma Gandhi University of Medical Sciences & Technology, Jaipur, India; 3 Department of Otorhinolaryngology, Pontificia Universidade Catolica do Rio Grande do Sul, Porto Alegre, Brazil; 4 Department of Otorhinolaryngology, University of Santo Tomas, Manila, Philippines; 5 Department of Neurology, Ludwig-Maximilians-Universitat Munchen, Munich, Germany

**Keywords:** Dix Hallpike test, Epley, nystagmus, otoconia, positional vertigo, vestibular

Peripheral paroxysmal positional vertigo (PPPV)—a termh preferred over benign paroxysmal positional vertigo (BPPV)—is the most common peripheral vestibular disorder encountered in clinical practice, yet its evaluation continues to reveal considerable variability across patient groups, testing conditions, and pathophysiological contexts (Alolayet and Murdin). This Research Topic covers a broad range of aspects of this highly relevant disease, including the influence of comorbidities on PPPV.

A systemic review by Alolayet and Murdin examines how metabolic, cardiovascular, psychological, and otologic comorbidities interact with PPPV and its treatment. Co-morbidities such as low Vitamin D levels, head trauma, migraine, several inner ear diseases (1), hypertension, and high cholesterol, are often associated with the lower success rate of repositioning maneuvers. Recognizing these associations is important for the prognosis, intensity of treatment and thereby also consultation of such patients. Clinical complexity may arise from patient-specific factors like age. Li et al. study depicted that elderly patients with presence of atypical symptoms of PPPV experience delays in diagnosis compared with younger individuals. Their findings describe how comorbidities may conceal the classical positional triggers of PPPV in aging populations. In fact, it is now advocated by The World Falls Guidelines: all older adults (>60 years) with objective or subjective balance problems, irrespective of symptomatic complaint, should have positional testing to examine for PPPV (2).

A retrospective study conducted by Lu et al., showed that timing of clinical assessment of BPPV appeared to be a key component of diagnostic variability. Their study demonstrated that positional tests yield a significantly higher rate of positive diagnostic findings in the morning compared with the afternoon. This is theoretically logical because it its assumed that the crystals form a agglomerate overnight while resting which has a higher impact on endolymphatic flow than single crystals (3). Clinicians should therefore consider the timing of testing during the day, particularly in patients with fluctuating symptoms.

Variability in head orientation and movement during positional maneuvers also introduces significant diagnostic uncertainty (Hentze et al.). Even minor deviations from standardized head positions can alter the intensity and direction of nystagmus by modifying canal stimulation, thereby reducing diagnostic accuracy. Simulation studies further demonstrate that both the initial position of otolithic debris and the sequence of positional testing can influence nystagmus direction and lead to altered interpretations of positional tests (4). Consistent with these findings, substantial variability in head angulation during commonly performed maneuvers such as the Epley maneuver has been documented, potentially contributing to inconsistent diagnostic and therapeutic outcomes (5). Collectively, these data underscore the critical role of precise technique, namely orientation of the canal tested in relation to the gravitational vector, test sequencing (one should begin with the horizontal canals, then posterior and finally the anterior) (6) and objective guidance in optimizing the reliability of positional testing.

Complementing these observations, Zuma E Maia et al. provided a neurophysiological perspective on horizontal canal PPPV (HC-PPPV) by emphasizing that correct side for maneuver selection is essential for effective treatment. In cases where the Supine Roll Test (SRT) shows minimal differences in nystagmus intensity, their algorithm combines SRT variant identification (geotropic vs. apogeotropic) with Bow- and Lean-Test responses. In practice, bowing identifies the affected side in geotropic HC-PPPV, whereas leaning does so in apogeotropic cases. Combining the results of both test findings using video-oculography recordings can enhance diagnostic results.

Xing et al. highlight diet as a modifiable risk factor in PPPV, proposing that dietary patterns may influence inner-ear health via antioxidant effects, improved microcirculation, and metabolic regulation. Genetic variability likely modulates these effects, as differences in genes related to otoconial integrity, vestibular function, and metabolism may alter dietary response and PPPV susceptibility and prognosis.

Collectively, a web of interacting factors influences outcomes in PPPV like patient demographics, comorbidities, the timing of assessment and the precision of clinical manoeuvres. Rather than viewing these findings as separate observations, they should be considered as overlapping pieces of a broader clinical picture. By integrating these perspectives, it helps clinicians to refine diagnostic strategies, anticipate sources of variability, and better adapt care to individual patients.

Going forward, several avenues merit focused exploration. Prospective studies examining how systemic health, otoconial stability, and canal biomechanics relate to symptom variability may clarify why some patients deviate from expected patterns. Efforts to improve diagnostic consistency—through enhanced maneuver training, supportive positioning technologies, or optimized testing workflows—may help improve reproducibility. Further, dedicated investigations in older adults and patients with comorbidities, namely any other inner ear diseases, remain essential, as these groups often present with atypical or muted manifestations that challenge conventional diagnostic assumptions.

Taken together, these perspectives highlight the evolving understanding of why PPPV, though common, is not uniformly straightforward to evaluate. Integrating physiological insights with practical clinical considerations can support more precise, patient-centered diagnostic approaches and ultimately improve outcomes for individuals with positional vertigo.

Finally, from a pragmatic clinical point of view every patient with vertigo or dizziness—independent of patient history—should be examined for PPPV with the correct orientation of the canal examined relative to gravitational vector during every visit not to overlook this well-treatable most frequent peripheral vestibular disease; one should begin with the diagnostic maneuvers for horizontal, then posterior and finally anterior canals; and every patient needs follow-up examinations to check the efficacy of the specific treatment maneuvers.
